# Dynamic variations in and prediction of COVID-19 with omicron in the four first-tier cities of mainland China, Hong Kong, and Singapore

**DOI:** 10.3389/fpubh.2023.1228564

**Published:** 2023-10-10

**Authors:** Xiaohua Ni, Bo Sun, Zengyun Hu, Qianqian Cui, Zhuo Zhang, Hua Zhang

**Affiliations:** ^1^College of Public Health, Zhengzhou University, Zhengzhou, China; ^2^Shenzhen Institute of Advanced Technology, Shenzhen University Town, Shenzhen, China; ^3^Shenzhen Institute of Advanced Technology, Chinese Academy of Sciences, Shenzhen University Town, Shenzhen, China; ^4^Research Center for Ecology and Environment of Central Asia, Chinese Academy of Sciences, Urumqi, China; ^5^University of Chinese Academy of Sciences, Beijing, China; ^6^State Key Laboratory of Desert and Oasis Ecology, Xinjiang Institute of Ecology and Geography, Chinese Academy of Sciences, Urumqi, China; ^7^College of Mathematics and Statistics, Ningxia University, Yinchuan, China; ^8^College of Geography and Remote Sensing Sciences, Xinjiang University, Urumqi, China

**Keywords:** COVID-19, omicron, dynamic variations, ARIMA, prediction

## Abstract

**Background:**

The COVID-19 pandemic, which began in late 2019, has resulted in the devastating collapse of the social economy and more than 10 million deaths worldwide. A recent study suggests that the pattern of COVID-19 cases will resemble a mini-wave rather than a seasonal surge. In general, COVID-19 has more severe impacts on cities than on rural areas, especially in cities with high population density.

**Methods:**

In this study, the background situation of COVID-19 transmission is discussed, including the population number and population density. Moreover, a widely used time series autoregressive integrated moving average (ARIMA) model is applied to simulate and forecast the COVID-19 variations in the six cities. We comprehensively analyze the dynamic variations in COVID-19 in the four first-tier cities of mainland China (BJ: Beijing, SH: Shanghai, GZ: Guangzhou and SZ: Shenzhen), Hong Kong (HK), China and Singapore (SG) from 2020 to 2022.

**Results:**

The major results show that the six cities have their own temporal characteristics, which are determined by the different control and prevention measures. The four first-tier cities of mainland China (i.e., BJ, SH, GZ, and SZ) have similar variations with one wave because of their identical “Dynamic COVID-19 Zero” strategy and strict Non-Pharmaceutical Interventions (NPIs). HK and SG have multiple waves primarily caused by the input cases. The ARIMA model has the ability to provide an accurate forecast of the COVID-19 pandemic trend for the six cities, which could provide a useful approach for predicting the short-term variations in infectious diseases.Accurate forecasting has significant value for implementing reasonable control and prevention measures.

**Conclusions:**

Our main conclusions show that control and prevention measures should be dynamically adjusted and organically integrated for the COVID-19 pandemic. Moreover, the mathematical models are proven again to provide an important scientific basis for disease control.

## 1. Introduction

In late 2019, coronavirus disease 2019 (COVID-19), the disease caused by severe acute respiratory syndrome coronavirus 2 (SARS-CoV-2), was first reported; subsequently, a pandemic ensued and was characterized by person-to-person transmission and asymptomatic patients (1, 2). For at least half a year, COVID-19 rapidly spread worldwide over more than 200 countries and regions. To address the rapid spread of COVID-19, the World Health Organization (WHO) declared it a pandemic. As of March 17, 2023, 760,360,956 confirmed cases and 6,873,477 deaths from COVID-19 have occurred globally.^1^

In 2023, the COVID-19 pandemic is still far from over (3). The pattern of COVID-19 will present as a mini-wave rather than a seasonal surge (4). During the past few years of the pandemic, because of virus mutations in special populations (e.g., older individuals, infants and populations with basic diseases), COVID-19 has been constantly transmitted worldwide (5, 6). The SARS-CoV-2 variants include the alpha variant of concern (B.1.1.7), beta variant (B.1.351), gamma variant (P.1), delta variant (B.1617.2) and Omicron variant (B.1.1.529), all of which are characterized by changes in transmission speed and virus virulence (7–9).^2^ Since the first discovery of the omicron mutant strain in South Africa in November 2021, it has spread rapidly worldwide and become the dominant strain in many countries and regions (7). The reduced pathogenicity and virulence of omicron mutant strains, characterized by an analysis of hospitalized patients during the peak of omicron infection in South Africa, showed a shortened incubation period in the population, mostly asymptomatic and mild cases, and a reduced risk of hospitalization and death (10). The immune escape ability is rapidly increasing, and omicron can escape more than 85% of the 247 human neutralizing antibodies screened. Different mutations have different effects on different classes of antibodies (11). The omicron strain significantly increases the risk of reinfection, which predicts that more susceptible individuals in the population with the omicron strain may cause a larger peak in the outbreak. Faster transmission, easier transmission through surfaces and aerosol media, and the omicron variant can exhale large amounts of neo coronavirus aerosol from patients themselves (12).

Due to the huge impacts that COVID-19 exerted on social economics and human lives worldwide, a large number of prevention and control measures have been employed in different countries (7, 13–15). Because the accurate simulation and prediction of infectious diseases can provide the scientific basis for adopting reasonable measurements, many COVID-19 models have been established to investigate disease variations and related impact factors and to predict future trends (16–19). Among these models, ARIMA and SARIMA models have been widely applied to analyze the linear, nonlinear and seasonal characteristics of COVID-19 (20–22). Gaetano Perone compared the use of the ARIMA, ETS, NNAR, TBATS, and hybrid models for predicting the second wave of COVID-19 hospitalizations in Italy, and the results demonstrated that hybrid models can better capture linear, nonlinear and seasonal models (23, 24) and accurately predict the risk of ending of a new coronary pneumonia outbreak and a second rebound using the SARIMA model (24). The ARIMA model is useful for the prediction of COVID-19 in Brazil and India and can also provide a reference for epidemic prevention and control and policy development in other countries (25).

Variations in the pandemic have led to changes in the control and prevention measures in different countries or cities over time. Due to multiple different complex factors, such as socioeconomic factors (e.g., population transmission, gross domestic product and medical resource level), control and prevention measure factors (e.g., nonpharmaceutical interventions (NPIs) and vaccines), and natural environmental factors (e.g., temperature, precipitation and humidity), the corresponding COVID-19 time series display different temporal characteristics (e.g., linear trend and multiple waves). In other words, analyzing and exploring the temporal variations in COVID-19 can illustrate the background impact factors in different countries and cities. Therefore, it is very important and significant to compare trends in the COVID-19 pandemic between different countries and cities; such comparisons can reveal efficient and valuable control and prevention measures for fighting future infectious diseases. Wang (26) compared dynamic variations in COVID-19 using a general disease dynamic model across 88 global countries encompassing population transmission (i.e., contact rates), disease detection capacity (i.e., detection rates) and immune loss rates. Using an innovative approach named “Yi Hua Jie Mu,” they identified one of the key parameters to predict the future changes in COVID-19 and compared the differences among countries with different climate regions. Chen (2) performed a cross-country core strategy comparison in four different countries (i.e., China, Japan, Singapore and South Korea) in the early period of the COVID-19 pandemic. China, Singapore, and South Korea adopted the containment strategy, and Japan adopted the mitigation strategy. Their results showed that the mitigation strategy was inferior to the containment strategy (27).

However, there are only a few comprehensive analyses of the whole COVID-19 period from 2020 to 2022. In particular, the studies comparing COVID-19 between different cities are limited, especially in the Omicron transmission period. Therefore, in this study, we focus on six different cities or countries with different cultures, population structures and control and prevention measurements, including Beijing (BJ), Shanghai (SH), Guangzhou (GZ), Shenzhen (SZ), Hong Kong (HK) and Singapore (SG). The research questions were as follows. (1) What are the transmission characteristics of the six cities during the period of 2020–2022, especially in the Omicron transmission period? (2) Does a time series analysis model exist to simulate and predict COVID-19 transmission with Omicron virus?

To address the above questions, we will first comprehensively explore the transmission characteristics of COVID-19 in the six cities during the period of January 2020–December 2022. Then, using the autoregressive sliding average method to predict the trend of the new coronavirus pneumonia epidemic can effectively use historical cases to build a prediction model, which has the characteristics of strong short-term prediction ability and simple operation. ARIMA models are widely used for the prediction and early warning of infectious diseases. The ARIMA model will be employed to simulate and predict the disease transmission trends for the six cities during the Omicron periods, which can lead to a better understanding of the different variations and provide important scientific bases for research on other infectious diseases.

## 2. Datasets and methods

### 2.1. Datasets used in this study

In this study, the datasets of the six cities are composed of socioeconomic datasets and population datasets from the period of 2012–2021 and COVID-19 pandemic datasets from the period of 2020–2022. The socioeconomic datasets include the GDP and medical resource level, and the population datasets include the total population, population transmission, population with ages smaller than 18 years and larger than 60 years, which are downloaded from the National Bureau of Statistics of China,^3^ Census and Statistics Department, Hong Kong, China^4^ and the Department of Statistics Singapore.^5^

The COVID-19 pandemic datasets of the six cities include the daily new confirmed cases, cumulative confirmed cases, imported cases and cumulative removed cases from 2020–2022. The disease datasets of the five cities (i.e., Beijing: BJ, Shanghai: SH, Guangzhou: GZ, Shenzhen: SZ and Hong Kong: HK) are sourced from the National Health Commission of the People’s Republic of China,^6^ and the Singapore COVID-19 datasets are from the World Health Organization.^7^

### 2.2. Methods

Autoregressive integrated moving average (ARIMA) models were developed by Box and Jenkins (28) and are widely used time series models for simulation and prediction studies (29–33). The basic form of the ARIMA model is ARIMA (*p*, *d*, *q*), where the nonnegative integers *p* and *q* are the orders of autoregressive and moving average polynomials, respectively, and *d* is the nonseasonal differencing required to make data stationary. An ARIMA (*p*, *d*, *q*) model can be expressed using lag polynomial L as the following equation:


$$\left(1-\overset{p}{\underset{i=1}{\sum }}\varphi_{i}L^{i}\right){\left(1-L\right)}^{d}=\left(1+\overset{q}{\underset{j=1}{\sum }}\theta_{j}L^{j}\right)\varepsilon_{j}$$

where $\varepsilon_{j}$ is a random error at time j , $\varphi_{i}$ , and $\theta_{j}$ are the coefficients.

In general, the ARIMA model can capture both nonseasonal and seasonal patterns of time series. There are three steps to forecast the time series: model identification, parameter estimation, and diagnostic checking of the model. In the first step of model identification, the stationarity and seasonality of the time series are determined, which need to be modeled before parameter estimation. The augmented Dickey-Fuller (ADF) test is used to detect whether the time series is stationary. If the *p* values of the ADF test are less than 0.05, the time series is stationary. If the time series is nonstationary, an autocorrelation function (ACF) plot is used to judge it as stationary with the differencing transformation, and the parameter d is determined. Seasonality can be obtained by taking seasonal differencing and regenerating ACF and partial autocorrelation function (PACF) plots.

For ARIMA model identification, ACF and PACF plots are also helpful to determine the values of the parameters of *p* and *q*. The most commonly used method (i.e., maximum likelihood) is employed to estimate the parameters of the appropriately selected model. In the end, the overall adequacy of the model is checked by the Ljung and Box test (34). In this study, the R 4.0.5 version is applied to construct the ARIMA model for simulating and predicting the COVID-19 pandemic time series. Generally, for the simulation of the time series models, the time series period will be divided into two parts which are the training data with the 70 percentages of the whole data and the testing data with the residual 30 percentages. In the ARIMA (*p*, *d*, *q*) model, 70% of the time series are selected as the training set, and the residual 30% of the time series are the test set for the six cities which are same as our previous study (13).

To assess the model’s performance, the correlation coefficient (CC), absolute error (AE), root mean square error (RMSE) and distance between indices of simulation and observation (DISO) are employed as in previous studies (7, 13, 26, 35, 36). The DISO index is a comprehensive assessment of multiple models, which is a merge of different statistical metrics with dimensions from 1 to n (37–39). In 2022, it was formally named CCHZ-DISO, which can be readily and widely applied to any subject of science due to its simplicity and flexibility advantages (38). The DISO equation is provided as follows.

For two time series, A and B, with a length of n, we assume that the observation time series is *A* = (a_1_, a_2_,…, a_n_), and the simulated time series is *B* = (b_1_, b_2_,…, b_n_). Then, the evaluated metrics CC, AE, RMSE, and DISO have the following forms.


$$\begin{array}{c} CC=\frac{\sum_{i=n}^{i=1}\left(a_{i}-\bar{a}\right)\left(b_{i}-\bar{b}\right)}{\sqrt{\sum_{i=n}^{i=1}{\left(a_{i}-\bar{a}\right)}^{2}}\sqrt{\sum_{i=n}^{i=1}{\left(b_{i}-\bar{b}\right)}^{2}}},\\ \mathrm{AE}=\frac{1}{n}\overset{i=1}{\underset{i=n}{\sum }}\left(b_{i}-a_{i}\right),\\ \mathrm{RMSE}=\sqrt{\frac{1}{n}\overset{i=1}{\underset{i=n}{\sum }}{\left(b_{i}-a_{i}\right)}^{2}}, \end{array}$$

$$DISO=\sqrt{{\left(CC-1\right)}^{2}+NAE^{2}+NRMSE^{2}}$$

where NAE and NRMSE are normalized by the averaged values of the observed time series.

## 3. Results

### 3.1. Temporal characteristics of COVID-19 in four first-tier cities

**Figure 1:**
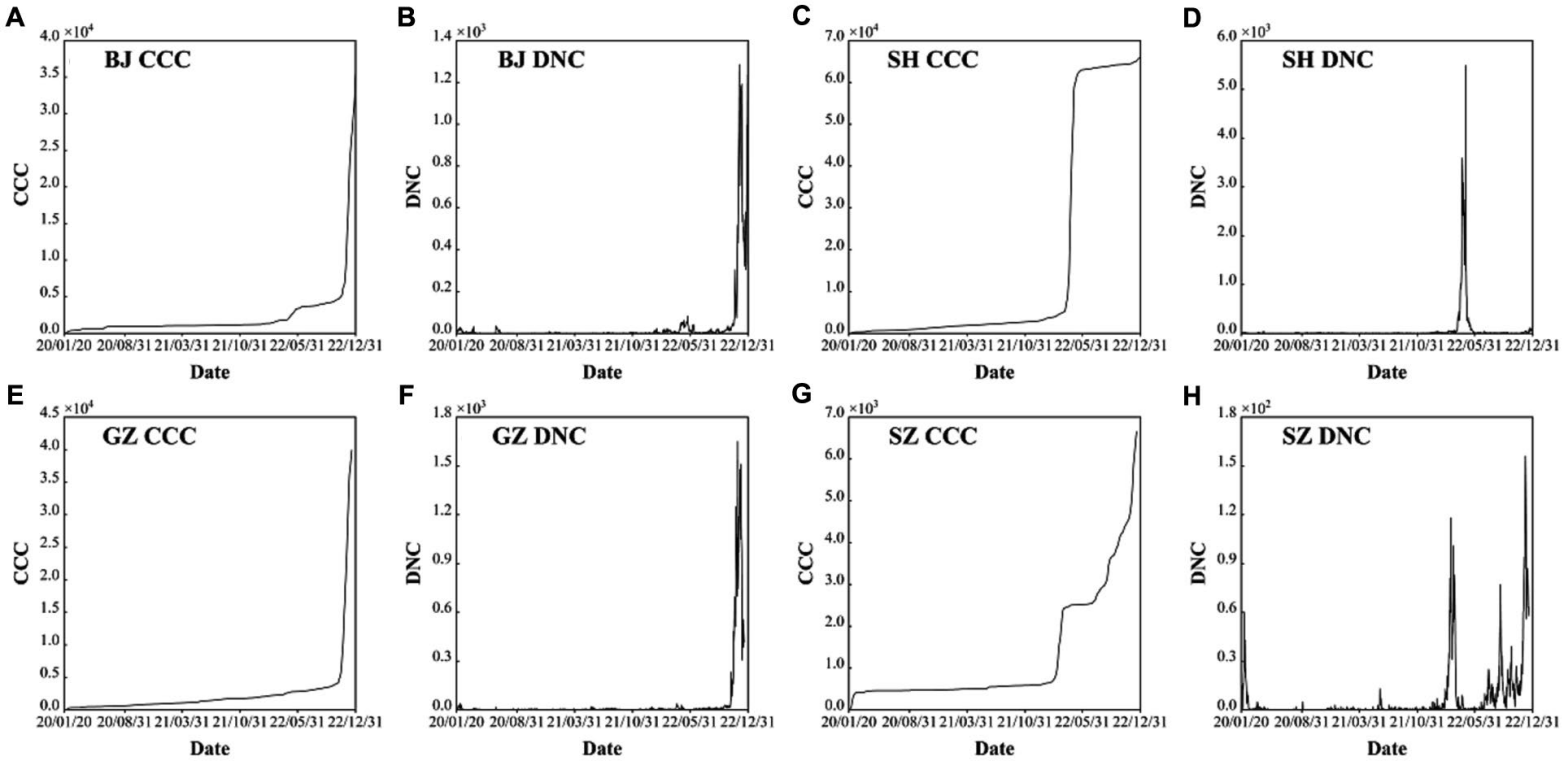
Confirmed cumulative cases (CCC) and daily new cases (DNC) of the four first-tier cities of mainland China: Beijing (BJ) **(A)** and **(B)**, Shanghai (SH) **(C)** and **(D)**, Guangzhou (GZ) **(E)** and **(F)**, and Shenzhen (SZ) **(G)** and **(H)** during the period of 2000–2022.

During the COVID-19 years from 2020 to 2022, the control and prevention measures of mainland China focus on “to prevent the coronavirus from spreading within the city/region or beyond,” “to prevent the coronavirus from re-entering the country to cause a new epidemic,” and the “Dynamic Zero COVID-19 Strategy,” which mainly include lockdown, wearing masks, COVID-19 vaccines and social distancing. After the three-year fight against COVID-19, mainland China has accumulated rich experience, which can be employed in the control and prevention of similar infectious diseases in the future. According to the COVID-19 pandemic development trends of the four first-tier cities of mainland China, each has its own variation characteristics (Figure 1).

**Figure 2:**
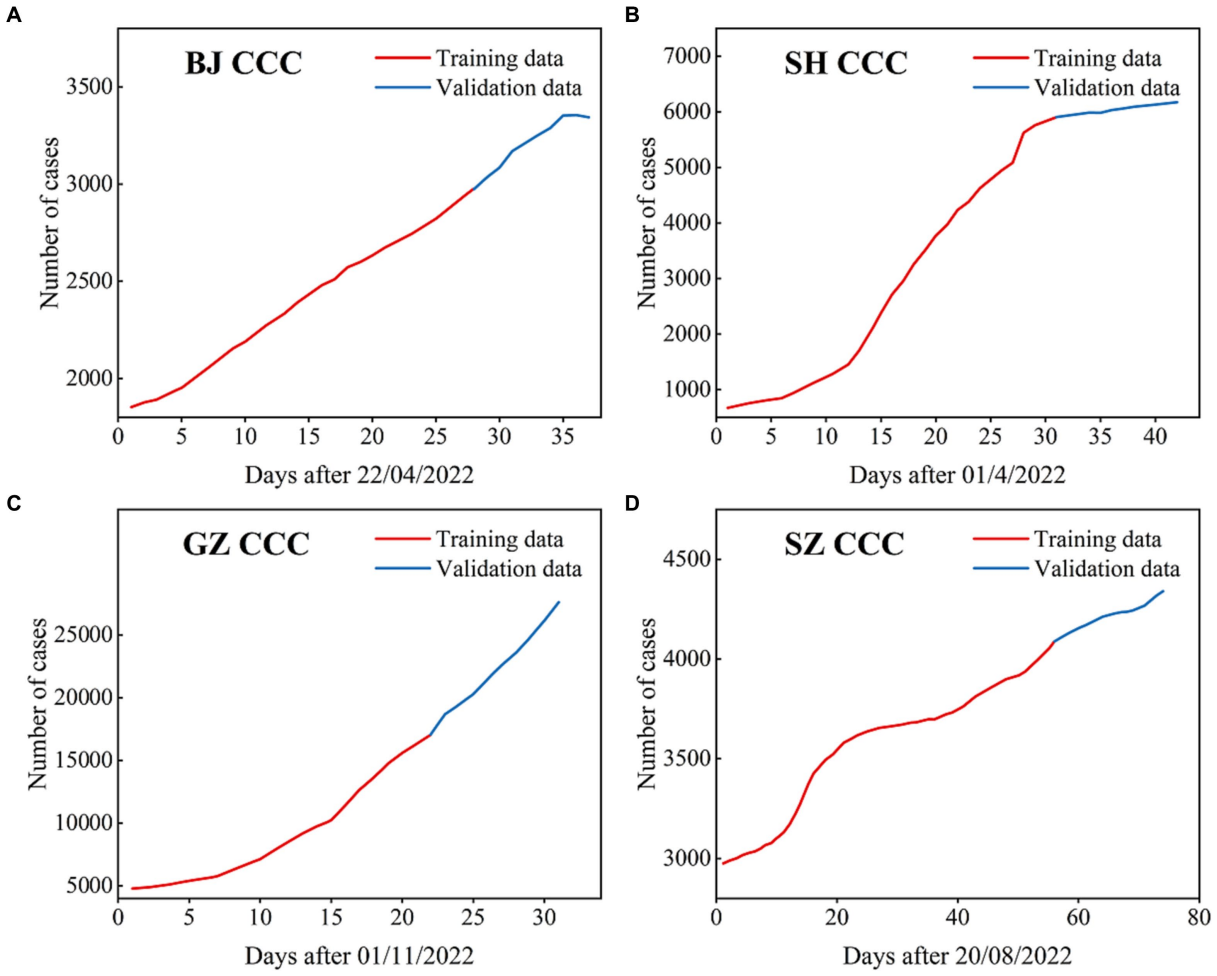
The CCC (confirmed cumulative cases) used in the ARIMA models for BJ **(A)**, SH **(B)**, GZ **(C)**, and SZ **(D)**, where the data with black lines are the training set and the data with blue lines are the test set.

As the capital of China, the COVID-19 trend BJ has a general stable variation on most days except for the outbreak caused by the omicron (Figures 1A,B). There are about six mini-waves of the COVID-19 with the largest daily new confirmed case number of 1,285 only in the sixth wave in November 29, 2022 (Table 1). Specifically, after the five local confirmed cases were observed on January 20th, 2020, the COVID-19 trend in BJ entered the early stage of local diffusion in early February. The pandemic had a stable situation, and the largest daily confirmed caseload was 36, which could not lead to the development of a large wave of COVID-19 cases. On January 15th, 2022, the first local omicron case was confirmed in BJ. Then, an increased wave was observed from April to May. Therefore, for BJ, the time period from April 22nd, 2022, to May 28th, 2022, is selected for the ARIMA model and analyzed in the following section (Figure 2A).

**Table 1:** The statistical characteristics of the epidemic waves of the six cities during the period of 2020–2022.

Epidemic wave		BJ	SH	GZ	SZ	HK	XG
First	Period	2020.1.20–2020.2.22	2020.1.20–2020.2.17	2020.1.24–2020.2.16	2020.1.24–2020.2.17	2020.2.16–2020.4.20	2020.2.15–2020.10.3
	Duration	33	27	23	24	65	232
	Max	29	27	38	60	82	1,426
	Max date	2020.2.2	2020.1.30	2020.2.1	2020.1.31	2020.3.29	2020.4.20
	Start to max	13	10	8	7	42	65
Second	Period	2020.2.26–2020.3.31	2020.3.12–2020.4.19	2021.5.26–2021.6.18	2022.2.12–2022.3.30	2020.6.16–2020.10.14	2021.8.23–2021.12.20
	Duration	34	38	23	46	120	119
	Max	32	52	18	71	149	5,324
	Max date	2020.3.23	2020.4.19	2021.5.30	2022.3.16	2020.7.30	2021.10.27
	Start to max	26	38	4	32	44	65
Third	Period	2020.6.11–2020.7.6	2022.2.16–2022.6.5	2022.4.2–2022.5.9	2022.8.23–2022.9.18	2020.10.15–2021.5.27	2021.12.21–2022.5.2
	Duration	26	109	37	26	224	132
	Max	36	5,489	39	69	115	26,032
	Max date	2020.6.13	2022.4.28	2022.4.13	2022.9.3	2020.11.29	2022.2.22
	Start to max	2	71	11	11	45	63
Fourth	Period	2022.1.10–2022.2.8		2022.10.2–2022.12.17	2022.11.9–2022.12.17	2021.12.8–2022.5.23	2022.5.3–2022.6.16
	Duration	29		76	38	166	45
	Max	23		1,645	146	79,876	6,442
	Max date	2022.1.29		2022.11.23	2022.12.4	2022.3.3	2022.5.18
	Start to max	19		52	25	85	15
Fifth	Period	2022.4.17–2022.6.29				2022.5.24–2023.1.28	2022.6.17–2022.8.29
	Duration	73				249	73
	Max	83				29,207	16,870
	Max date	2022.5.22				2022.12.31	2022.7.13
	Start to max	35				221	26
Sixth	Period	2022.10.1–2022.12.4					2022.8.30–2022.1.6
	Duration	64					129
	Max	1,285					11,934
	Max date	2022.11.29					2022.10.18
	Start to max	59					49

As the most important foreign trade center in mainland China, SH had the largest population transmission. Except the low-level oscillation in most times, only three epidemic waves are observed with the largest daily new case number of 5,489 in April 28th, 2022 (Table 1). The first observed COVID-19 case in SH was observed on January 20, 2020. At the early stage, some confirmed cases were reported each day in SH. The outbreak of COVID-19 in SH was observed on March 1st, 2022, and the local daily caseload increased from 96 to 326 on March 29th (Figure 1D). In April, the COVID-19 pandemic in SH became highly developed with respect to confirmed cases, and the number of asymptomatic cases rapidly increased. From the end of March to May 12th, the local cumulative number of cases was more than 60,000, which is approximately four times the total confirmed cases in China in 2021 (15,243 cases). However, the more concerning aspect is the drastic increase in the number of asymptomatic cases, with a number exceeding 1,000 after March 24th. During the period from April 4th-April 26th, the daily number of new asymptomatic cases was as high as 10,000 for 20 consecutive days. As a super large international city, SH had the most input confirmed cases among all of China. Moreover, most of the confirmed cases were caused by the Omicron BA.2 and BA.2.2 variants; these variants cause more than 4 times the number of case than the Delta variant. Based on the transmission characteristics of the Omicron variant in SH, the time period from April 1st, 2022, to May 12th, 2022, will be examined in the ARIMA model (Figure 2B).

In GZ, COVID-19 infectious diseases continuously increased from January 2020 to November 2022. In this period, there are four waves (Figures 1E,F). After November 2022, the COVID-19 outbreak increased. Combined with the high population density, high population transmission and complex spread chains, the infection disease diffusion risk and the difficulty of its control increased significantly. In the latest outbreak, the largest daily new case number is 1,645 at November, 23, 2022 (Table 1). According to the transmission characteristics in the 3 years, the COVID-19 time series from November 1st, 2022, to December 1st, 2022, will be examined in the ARIMA model (Figure 2C).

SZ has 16 ports and is thus one of the most entry-exit cities in China. As an economic center and international city, it attracts a large number of young people. Overall, during the COVID-19 period of 2020–2022, there are four epidemic waves: (1) the first wave is from January, 24, 2020 to February, 16, 2020 with the largest daily new confirmed cases value of 60; (2) the second wave is from February, 12, 2022 to March, 30, 2022 with the largest daily new confirmed cases value of 70; and (3) the third wave is from August, 23, 2022 to September, 18, 2022 with the largest daily new confirmed cases value of 69; and the fourth wave is from November, 9, 2022 to December, 17, 2022 with the largest daily new confirmed cases value of 146 (Figures 1G,H; Table 1). After the first wave was brought under control, nearly 600 days passed with only sporadic increases and no widespread epidemic. The interval between the second wave and the third wave was short, and there were local small wave variations under most situations, which is similar to most mainland cities. Due to its high rate of young people, the death rate was small. The COVID-19 Omicron wave started on July 12th, 2022. In this study, the disease transmission period from August 20th, 2022, to November 1st, 2022, will be examined in the ARIMA model (Figure 2D).

### 3.2. Temporal characteristics of COVID-19 in HK and SG

**Figure 3:**
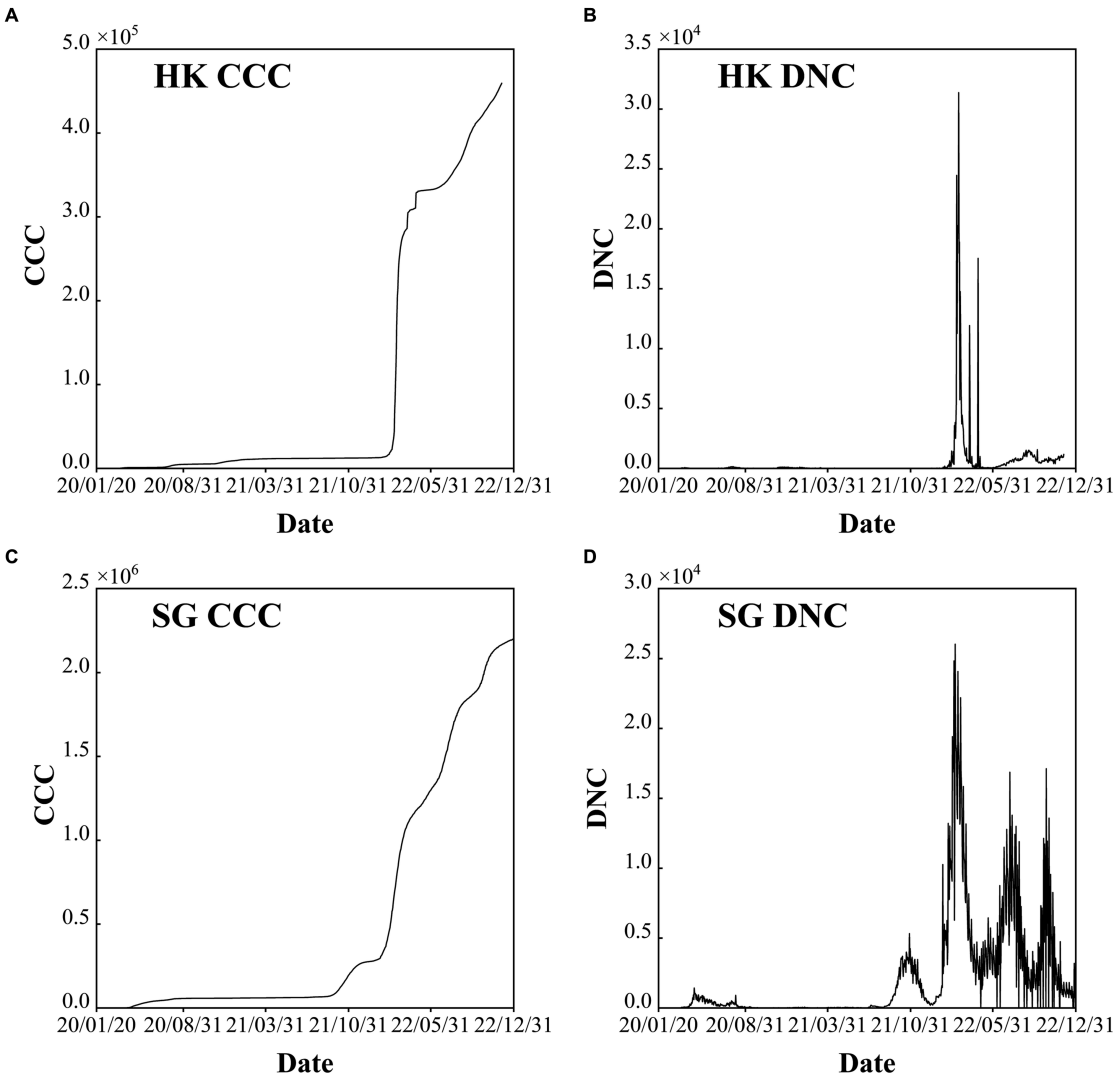
Confirmed cumulative cases (CCC) and daily new cases (DNC) of Hong Kong (HK) China **(A)** and **(B)**, and Singapore (SG) **(C)** and **(D)** during the period of 2020–2022.

HK has a population of more than 7.33 million and is one of the regions with the highest population density, with a land area of 1,113 km^2^. There are five epidemic waves with the largest daily new confirmed cases value of 79, 876 in March 3rd, 2022 (Table 1). On January 23rd, 2020, the first COVID-19 case was detected in Hong Kong, and then, the disease was transmitted in the following years. In general, Hong Kong has experienced five waves of the COVID-19 pandemic in the past 3 years (Figures 3A,B). The first wave lasted for approximately 1 month, and the second wave lasted for approximately 2 months. The third wave started in early November 2020 and lasted for no more than 2 months. In January 2021, the fourth wave was an outbreak lasting at least 5 months. Compared to the fifth wave, the first four waves had few confirmed cases, and the COVID-19 pandemic was still stable, mainly due to the “dynamic COVID-19 zero” strategy.

However, after the number of confirmed cases was out of control at the end of 2021, the COVID-19 Omicron variant began to circulate, and it spread to the whole society after only 1 month. The severe fifth wave started in February 2022, and the daily number of new confirmed cases reached its maximum in March before decreasing to 200–300 cases in May. Subsequently, the COVID-19 pandemic fluctuated. Since September 26th, 2022, the daily new confirmed cases have been approximately 5,000 since the adjustment of the control and prevention measures (Figure 3B).

From 2020 to 2022, SG has experienced four stages, each highlighting the global situation at the time as well as the country’s strategies to fight the COVID-19 pandemic. Six epidemic waves are observed with the largest daily new confirmed cases value larger than 1,200 (Table 1). The four stages are (1) early days of fog (January–March 2020), (2) fighting a pandemic (April 2020–April 2021), (3) rocky transition (May 2021–November 2021) and (4) learning to live with COVID-19 (December 2021-Present) (https://www.gov.sg/article/covid-19-white-paper White Paper on Singapore’s Response to COVID-19: Lessons for the Next Pandemic) (Figures 3C,D). The total cumulative confirmed COVID-19 cases exceed 2 million, which accounts for approximately 50% of the total population (i.e., 5.45 million at the end of 2022). After the first confirmed cases were reported on January 23^rd,^ 2020, the pandemic started to spread in March. To fight against COVID-19, NPIs and COVID-19 vaccines were used in tandem. After reaching the projected goal of the vaccines, the number of confirmed COVID-19 cases largely increased with approximately five waves, and each wave persisted for approximately 3–4 months. The serious outbreak wave was caused by the COVID-19 Omicron variant, with 26,032 new cases daily. Adequate medical resources and a high vaccination rate play a key role in COVID-19 control in SG.

### 3.3. Simulation and prediction of COVID-19 in the four first-tier cities in mainland China

In this section, the cumulative number of confirmed COVID-19 cases in the four first-tier cities in mainland China are simulated and predicted by the ARIMA model. The time series periods of the four cities are as follows: from April 22nd, 2022 to May 2022 for BJ; from April 1st, 2022 to May 12th, 2022 for SH; from November 1st, 2022 to December 1st, 2022 for GZ; and from August 20th, 2022 to November 1st, 2022 for SZ (Figure 2). The training set is 70% of the total number of time series, and the remainder of the time series is the test set for all four cities.

**Figure 4:**
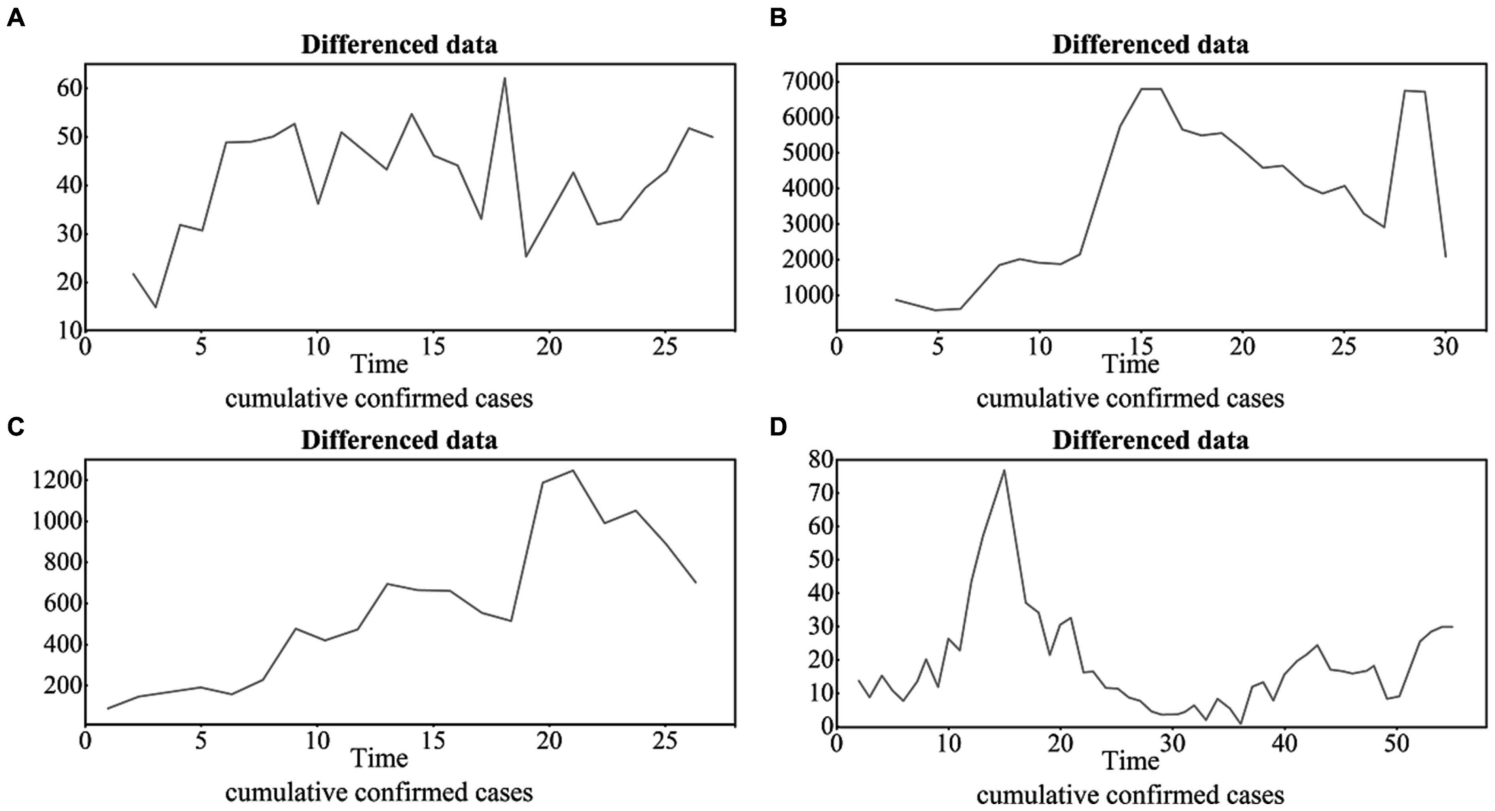
The differenced time series of the four cities with the difference orders of *d* = 1,2,2,1 for BJ, SH, GZ and SZ **(A)**, **(B)**, **(C)** and **(D)**, respectively.

According to the ADF test (i.e., white noise test and unit root test), the original time series of all four cities are not stationary, with ADF values and *p* values of (−2.3049, 0.4561) for BJ (−2.8156, 0.2594) for SH, (1.3957, 0.8026) for GZ, and (1.3957, 0.8026) for SZ. After the difference, the time series becomes stationary, and the difference orders are *d* = 1,2,2,1 for BJ, SH, GZ, and SZ, respectively (Figure 4).

**Figure 5:**
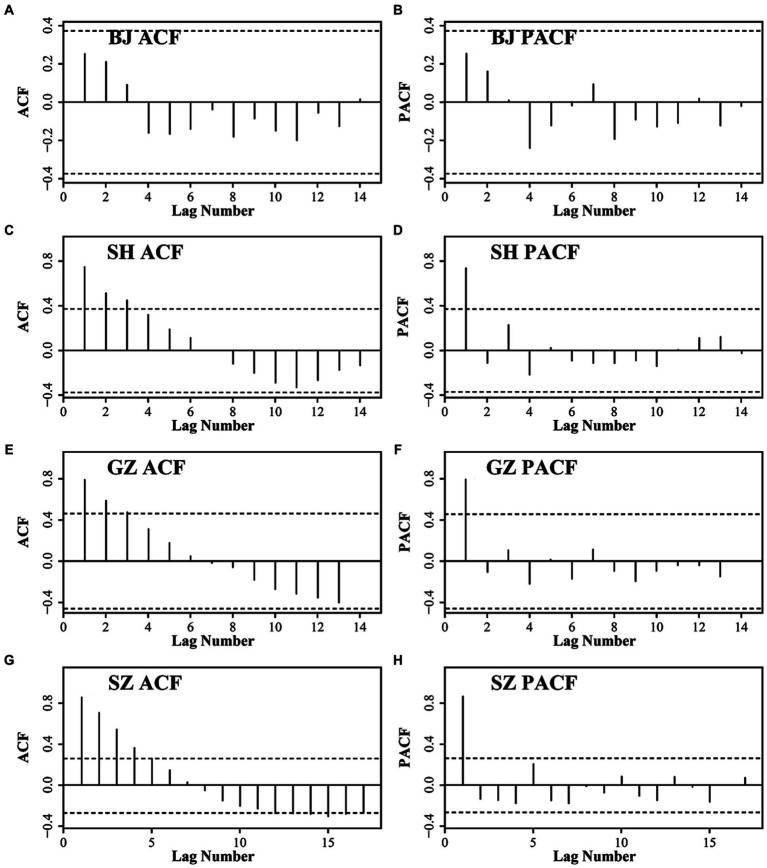
The ACF and PACF results for BJ **(A)** and **(B)**, SH **(C)** and **(D)**, GZ **(E)** and **(F)**, and SZ **(G)** and **(H)**.

According to the ACF and PACF results (Figure 5), the test from the low order to high order and the AIC criteria, the ARIMA models of the four cities are finally set as ARIMA (1,1,0), ARIMA (0,2,0), ARIMA (2,4,6), and ARIMA (1,1,0) for BJ, SH, GZ, and SZ, respectively.

**Figure 6:**
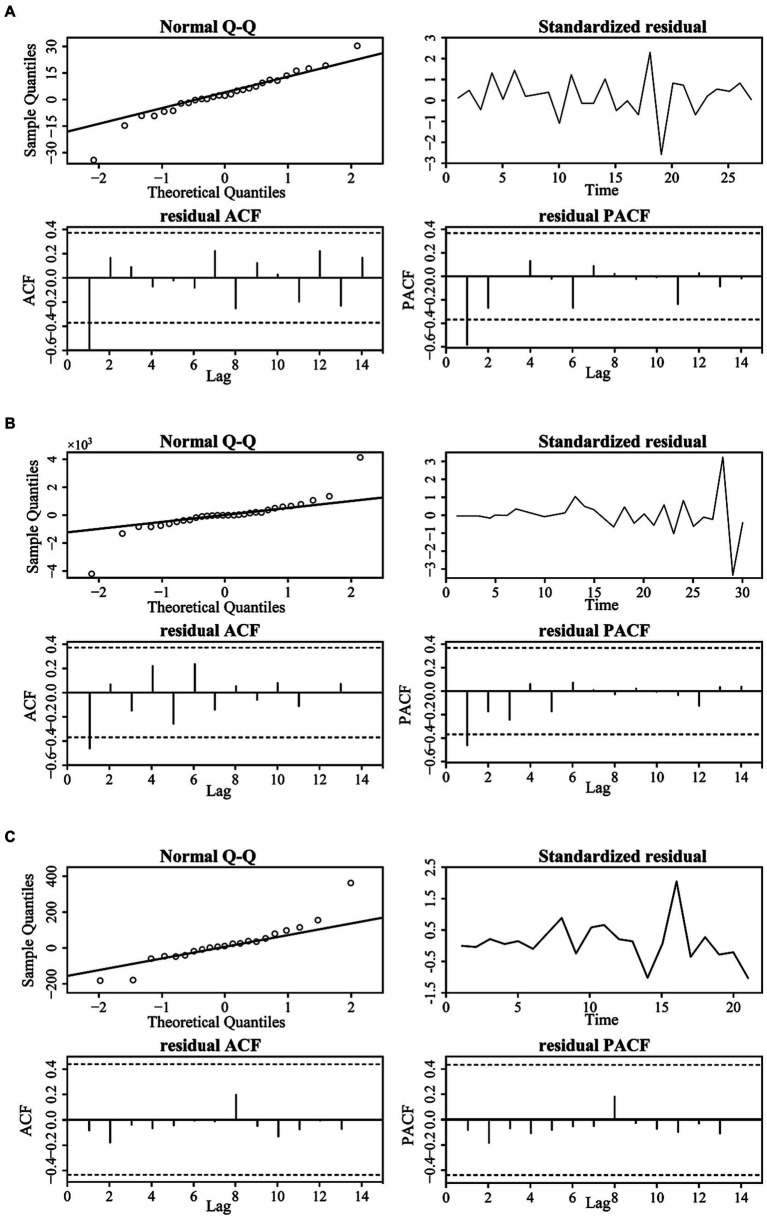

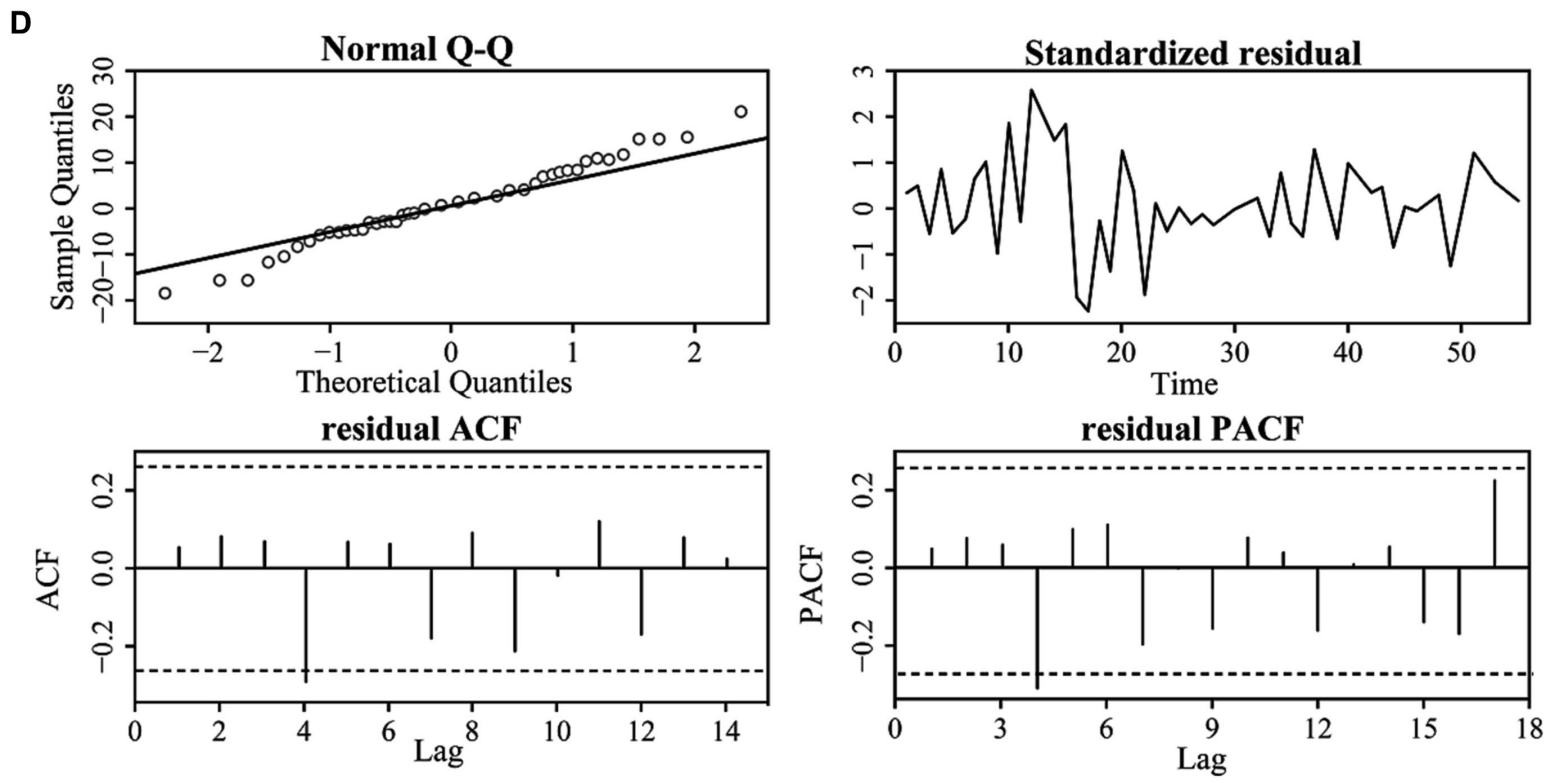
The residual correlation results of the original time series for the four cities: BJ **(A)**, SH **(B)**, GZ **(C)**, and SZ **(D)**.

Figure 6 displays the QQ plots of the residual time series; they are distributed around the diagonal line, which indicates that the residuals of the original are normally distributed. Moreover, the residuals are distributed around zero, and the autocorrelation and partial autocorrelation fall within the confidence interval, which indicates that the ARIMA model residuals are white noise time series, and we can use the ARIMA model to predict the COVID-19 variations.

**Figure 7:**
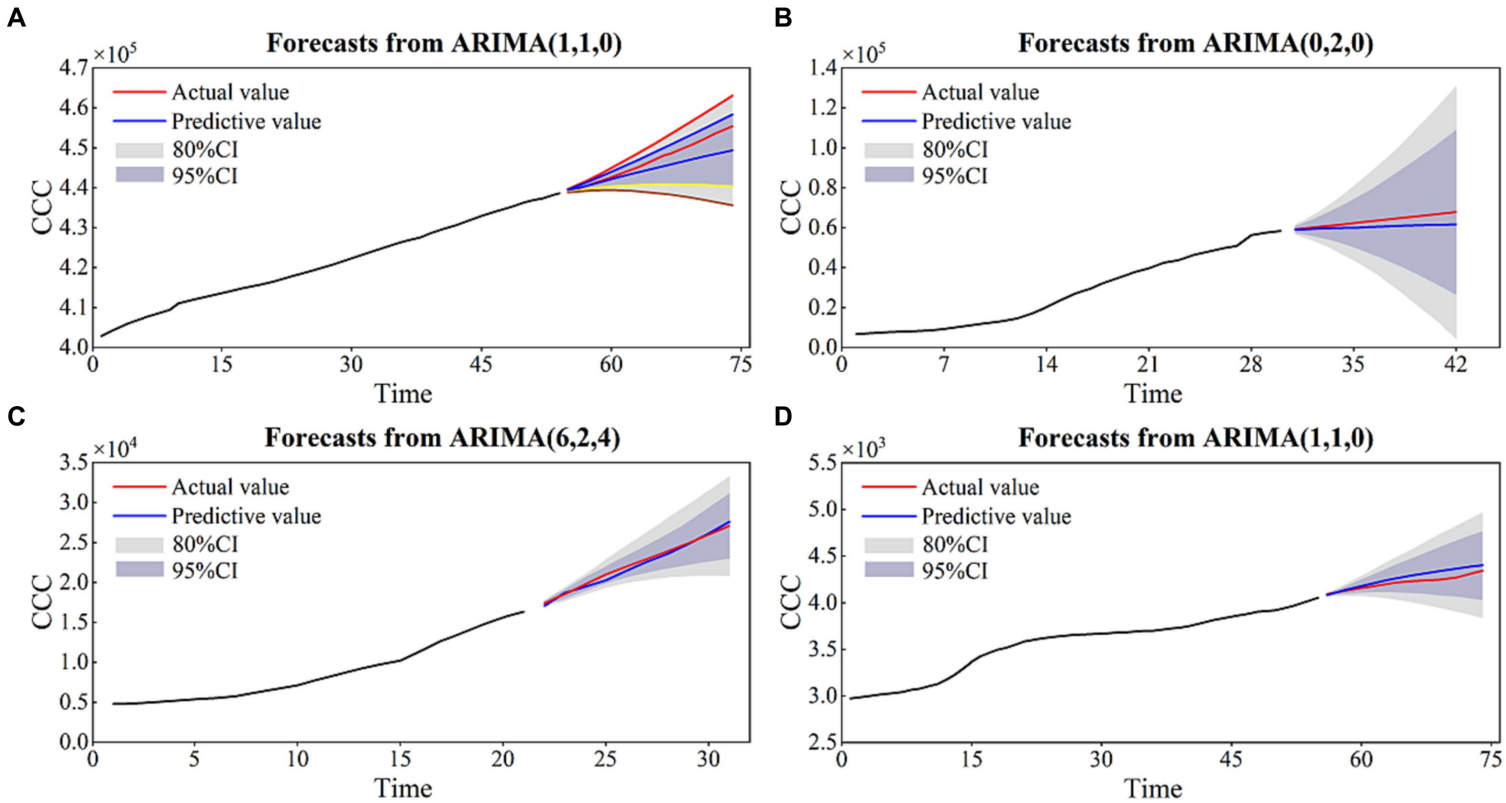
The prediction results of the ARIMA models for BJ **(A)**, SH **(B)**, GZ **(C),** and SZ **(D)**.

According to Figure 7 and the accurate evaluation results revealed by the CC, AE, RMSE, and DISO, the ARIMA models of the four cities can effectively capture the COVID-19 variations and have a high accuracy in predicting the cumulative confirmed cases (Table 2). Specifically, the CC values between the observed time series and the prediction time series are 0.99, 1, 1, and 0.98 for BJ, SH, GZ, and SZ, respectively. The DISO values are 0.03, 0.08, 0.02, and 0.03. The MAE values are −0.02, 0.05, 0.01, and 0.01 for the four cities. The NRMSE values are 0.02, 0.06, 0.01 and 0.01. The smallest relative error of BJ, SH, GZ, and SZ are −0.001, 0.001, 0.004, and − 0.0005 with the observation data vs. prediction data of 2,975 vs. 2,972, 59,068 vs. 59,128, 24,820 vs. 24,909 and 4,110 vs. 4,108 (Figure 7).

**Table 2:** The evaluation results of the ARIMA models for six cities/country.

City/Country	Time period		CC	MAE	RMSE	DISO
BJ	2022-5-19	2022-5-28	0.99	−0.02	0.02	0.03
SH	2022-5-1	2022-5-12	1.00	0.05	0.06	0.08
GZ	2022-11-22	2022-12-1	1.00	0.01	0.01	0.02
SZ	2022-10-14	2022-11-1	0.98	0.01	0.01	0.03
HK	2022-11-7	2022-11-26	1.00	0.00	0.01	0.01
SG	2022-7-29	2022-11-27	0.99	0.13	0.15	0.20

### 3.4. Simulation and prediction of COVID-19 in HK, China, and SG

**Figure 8:**
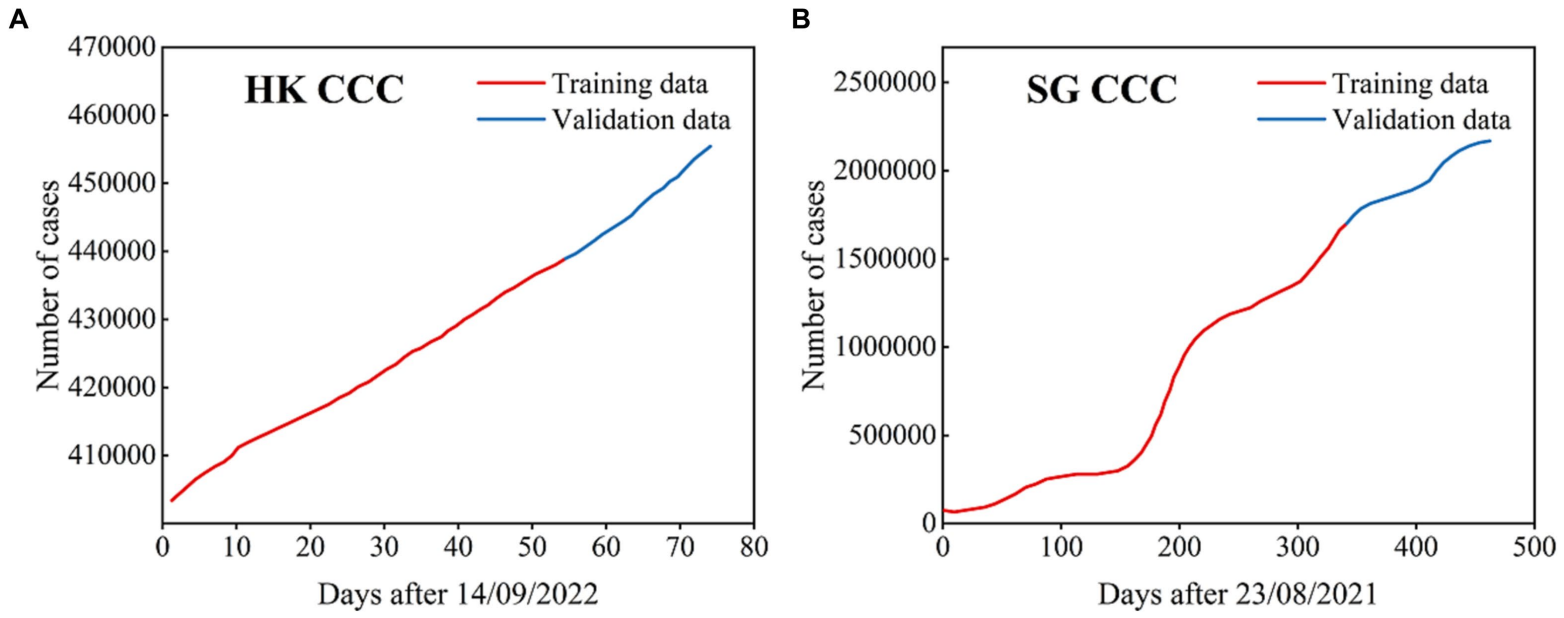
The cumulative confirmed COVID-19 cases used in the ARIMA models for HK **(A)** and SG **(B)**, where the data with black lines are the training set and the data with blue lines are the test set.

We used the ARIMA model to simulate and predict the cumulative number of COVID-19 cases in HK, China and SG. The corresponding COVID-19 periods focus on the Omicron transmission period, i.e., from September 14th, 2022, to November 26th, 2022, in HK and from August 23rd, 2021, to November 27th, 2022, in SG (Figure 8).

For HK, the total number of cumulative case time series is 74 days, where the cumulative cases over 54 days are the training set and the remaining time series are the test set. For SG, the total number of cumulative case time series is 462 days, where the cumulative cases over 340 days are the training set and the remaining time series are the test set.

**Figure 9:**
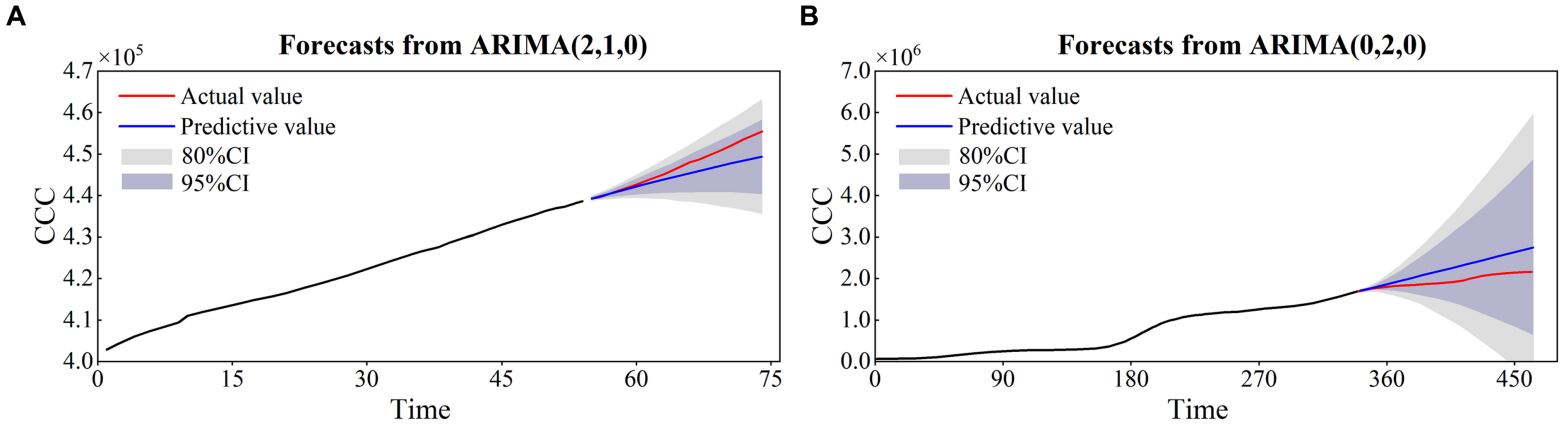
The prediction results of the ARIMA models for HK **(A)** and SG **(B)**.

After the ADF test, differenced time series, ACF and PACF test, the ARIMA models of HK and SG were finally determined to be ARIMA (2,1,0) and ARIMA (0,2,0). Using the ARIMA models, we obtained the prediction results of the cumulative confirmed cases for HK and SG, as shown in Figure 9. The temporal variations in the cumulative confirmed cases of HK and SG are well captured by the ARIMA models. The statistical metrics of CC, RE, RMSE and DISO are quantitatively evaluated for the models’ performances (Table 2). The CC values are 1 and 0.99, the NRE values are 0 and 0.13, the NRMSE values are 0.01 and 0.15, and the DISO values are 0.01 and 0.20 for HK and SG, respectively. For the overall performance, the DISO values are 0.01 and 0.2 for the two cities. The smallest relative errors of HK and SG are nearly to zero with the observation data vs. prediction data of 439,251 vs. 439,253 and 1,702,392 vs. 1,703,019 (Figure 9).

### 3.5. Comparison of COVID-19 in China and other countries

During the past 3 years, all the countries have their own COVID-19 transmission characteristics, and the special prevention and control strategies based on their COVID-19 pandemic variations and their social-economic characteristics (e.g., economic levels, medical resource level, population number). Hence, it is necessary to have a comparison of COVID-19 pandemic between China and other countries.

“Dynamic COVID-19 Zero” strategy and strict NPIs are through the whole COVID-19 pandemic prevention and control in China in the past years, which are the highly efficient measurements and make great contributions for the fight against the COVID-19 in the world. It is suggested that China, SG and South Korea with the containment strategy are better than Japan with the mitigation strategy (27). Although India adopted the containment strategy as China, a rapid increase in daily new cases and a high mortality were observed due to the unstrict measurements (40). Denmark and Norway employed the same COVID-19 strategy from containment to suppression and Sweden is from containment to mitigation (41). The mechanism of“Dynamic COVID-19 Zero” strategy is assessed by a compartmental model which highlights the importance and efficiency of the containment of the COVID-19 epidemic (42).

In fact, we should learn the lessons toward a more effective response to other public health emergencies in future. For example, a healthier and safer society requires that countries develop and employ the coherent and context-specific national strategy, which can improve the governance of public health emergencies, minimize fragmentation and tackle upstream structural issues (43).

## 4. Discussion

### 4.1. Differences in the COVID-19 pandemic transmission backgrounds of the six cities

In general, it is known that infectious disease transmission characteristics are caused by different complex environmental factors, including social environmental factors (e.g., GDP, population number, NPIs and medical resources) and natural environmental factors (e.g., temperature, precipitation, humidity, and wind). The COVID-19 pandemic variation trends of these six cities also result from complex environmental factors, which are the major backgrounds of disease transmission and outbreak and are under control. Moreover, the environmental factors differ across these six cities.

Specifically, the total populations of the six cities at the end of 2021 were 21.88 million for BJ, 24.89 million for SH, 18.81 million for GZ, 17.68 million for SZ, 7.41 million for HK, and 5.64 million for SG. The corresponding population densities of the six cities were 1,334 per km^2^, 3,925 per km^2^, 2,512 per km^2^, 6,484 per km^2^, 7,060 per km^2^ and 8,357 per km^2^, respectively. The percentages of the population aged 65 and over in the six cities were 14.2, 26.9, 13.33, 3.22, 19.56 and 12.03%, respectively. SH has the most severe aging, followed by HK; such aging leads to substantial challenges for COVID-19 control and prevention, especially during the omicron transmission period.

Vaccination can largely decrease the infection rate and severity rate and greatly reduce the mortality rate, especially for key populations (e.g., older adult people and those with underlying diseases such as COVID-19), which is one of the most efficient measures to control and prevent infectious diseases (44). As of January 2, 2023, the total number of COVID-19 vaccinations exceeded 3.47 billion, and 89.6% of the population older than 60 years will have received one vaccine dose. The COVID-19 vaccination rate in HK is as high as 94.4%. However, the very low vaccine rate of older adult people in HK caused a high mortality rate during the fifth wave. Singapore has one of the lowest mortality rates, which is due to its high vaccine rate and enriched medical resources (45). In the past three years, the total confirmed case number of SG was higher than 2.18 million, and the mortality number was only 1707, with a percentage of 0.0783%. In March 2022, 92% of the population received the vaccination, and more than 70% of the population received the booster dose in Singapore. The other contributors to the achievements in COVID-19 control and prevention in SG are highly efficient medical resource integration with patients, which is similar to China.

### 4.2. Mathematical models related to the simulation and prediction of COVID-19

Mathematical models can predict how infectious diseases progress to have the likely outcome of an epidemic and help to provide scientific information for public health interventions (26, 46, 47). Since the outbreak of the COVID-19 pandemic, mathematical models have become a crucial and important approach to accurately understand the transmission characteristics and mechanisms in controlling COVID-19 (48), which usually include time series models (e.g., generalized additive models, autoregressive integrated moving average models, and artificial neural network models) (49–51), and dynamic models (e.g., ODE: ordinary differential equation models, PDE: partial differential equation models and statistical equation models) (52–57).

Time series models have their own advantages in modeling the prevention and control of infectious diseases, with many featuring ease of use and fast performance for rapid diagnosis in the early stages of an outbreak. These models are less affected by parameter changes because they are driven by historical data and statistics. However, the natural transmission characteristics and clinical features of the disease are not considered in most time series models. The dynamic models derived by the ODE and PDE models are constructed according to the disease transmission mechanisms and diffusion characteristics in different types of populations. When the initial values and parameter values are set, the dynamical models have the advantages of assessing the nonlinear transmission characteristics, the interactions of the different populations and the contributions of the key parameters. However, some uncertainties will exist in the parameter estimation. Hence, hybrid models based on time series models and dynamic models will be a new trend in disease projection.

As one of the most widely used time series models, ARIMA is easily used and not strict on the time series, which usually can have an accurate short projection for the disease variations. During the COVID-19 pandemic period, there are numerous works about the simulation and prediction of this disease by time series models. ARIMA is one of the widely models in the COVID-19 analysis. For example, Ceylan (2020) suggested that ARIMA models are significant in predicting the prevalence of the COVID-19 pandemic (58). For some highly complex nonlinear time series, it is difficult to simulate and predict the disease variations by the ARIMA model. Therefore, in this study, we select the cumulative confirmed case time series to construct the ARIMA models for the six cities. For the daily new confirmed case time series or other infectious diseases, new models will be employed to explore the more complex characteristics of the diseases in our future research.

In addition, other time series models are also applied in simulating and predicting the COVID-19 variations (e.g., NARNN: nonlinear autoregression neural network; LSTM: long-short term memory). After the comparison of ARIMA, NARNN and LSTM, suggested that LSTM was the most accurate model (30). A family autoregressive models are used to analyze the real world time series data of confirmed and recovered COVID-19 cases based on two-piece scale mixture normal distribution (59). They found that the proposed algorithm outperforms other standard autoregressive time-series models. A hybrid deep learning method-Bayesian optimization model is applied to predict the COVID-19 confirmed cases (60). They pointed that the combination of multiple models can provide a more accurate prediction result than an individual linear or nonlinear model. Therefore, the hybrid models or the combinations of multiple models will provide efficient approaches to predict the disease time series. We will leave these for our other future analyses.

### 4.3. Two limitations of this study

The dynamical variations and predictions of the COVID-19 with Omicron in the six cities are comprehensively analyzed in this study. The basic characteristics of the COVID-19 transmissions are obtained. Moreover, the primary simulation and prediction of the cumulative confirmed cases are well explored by the ARIMA model. However, there are two limitations in this study which constrained the well understand of the COVID-19 transmissions.

The first limitation is the dataset. In general, the more datasets, the more characteristics of the COVID-19 can be obtained. In this study, daily new confirmed cases, cumulative confirmed cases, imported cases and cumulative removed cases of the six cities are used to reveal the COVID-19 transmission characteristics. But if other important datasets can be included the more detail characteristics of the COVID-19 will be obtained, which can help us to well understand the pandemic in the six cities. For example, the detail quarantine strategies and the wearing mask proportion datasets can provide detail information for the COVID-19 transmission and also provide important information for the simulation and prediction. The COVID-19 vaccine type, COVID-19 vaccine proportion and vaccination times play a key role for understanding the COVID-19 transmission and constructing the COVID-19 models.

The second limitation of this study is the simulation and prediction model. Although the ARIMA model can capture the characteristics of the cumulative confirmed cases and have accurate simulation and prediction, the daily new cases are not simulated and predicted. Therefore, two or more models will be employed to predict the other COVID-19 variables in the future study, such as the dynamic models constructed by the ODE and hybrid models by the dynamic models and time series models.

## 5. Conclusion

In this study, a comprehensive analysis of the COVID-19 pandemic variations in the four first-tier cities of mainland China, Hong Kong, China and Singapore from 2020 to 2022 was conducted. The population number, population density, and medical resources are discussed from 2012 to 2021, which are the major background of COVID-19 transmission. Moreover, a widely used time series model ARIMA is applied to simulate and forecast the COVID-19 variations in the six cities. The major conclusions are as follows.

1. The six cities have their own temporal characteristics, which are determined by the different control and prevention measures. The four first-tier cities of mainland China (i.e., BJ, SH, GZ, and SZ) have similar variations with one wave because of the same “Dynamical COVID-19 Zero” strategy and strict NPIs. Hong Kong and Singapore have multiple waves primarily caused by the input cases.
2. NPIs, high vaccination rates and highly efficient integrated multiple measures play key roles in controlling COVID-19 pandemic transmission, such as avoiding community communication and protecting vulnerable populations.
3. The ARIMA model has the ability to provide an accurate forecast of the COVID-19 pandemic tendency for the six cities, which could provide a useful approach to predict the short-term variations in infectious diseases. Accurate forecasting has significant value for adopting reasonable control and prevention measures.

Our main conclusions show that control and prevention measures should be dynamically adjusted and organically integrated for the COVID-19 pandemic. Moreover, the mathematical models are proven again to provide an important scientific basis for disease control. These are valuable experiences, and the knowledge accumulated in recent years can enable us to better fight against future infectious diseases in the world.

## Data availability statement

The original contributions presented in the study are included in the article/supplementary material, further inquiries can be directed to the corresponding author.

## Author contributions

ZH and XN: conceptualization, writing-review, and editing. ZH, XN, ZZ, QC, BS, and HZ: data curation. XN, ZH, ZZ, and BS: formal analysis. All authors contributed to the article and approved the submitted version.

## Footnotes

^1^https://covid19.who.int/

^2^https://www.who.int/en/activities/tracking-SARS-CoV-2-variants

^3^http://www.stats.gov.cn/sj/

^4^https://www.censtatd.gov.hk/sc/

^5^https://www.singstat.gov.sg/

^6^http://www.nhc.gov.cn/xcs/xxgzbd/gzbd_index.shtml

^7^https://covid19.who.int/data
